# Nanosurfer flash-mobs: electric-field-choreographed silver migration on graphene oxide†

**DOI:** 10.1039/c9na00151d

**Published:** 2019-04-11

**Authors:** Yong Han Jerome Leow, Patria Yun Xuan Lim, Sharon Xiaodai Lim, Jianfeng Wu, Chorng-Haur Sow

**Affiliations:** Dunman High School 10 Tanjong Rhu Road Singapore 436895; Department of Physics, National University of Singapore 2 Science Drive 3 Singapore 117542 physowch@nus.edu.sg +65 67776126 +65 65162957

## Abstract

We report for the first time the ability to direct and control the migration path of silver nanoparticles across graphene oxide (GO). With the help of a focused laser beam, we demonstrated choreographed nanoparticle assembly on GO *via* a directed electric-field. Silver migration and the resultant dendrite formation on GO were characterized through electrical testing coupled with fluorescence microscopy. The proposed mechanism for silver migration in GO involves the interlayer water between GO sheets serving as the electrolyte for the electrochemical process. This interlayer water facilitates the disappearance of dendrites through oxidation and dissolution into the water. Furthermore, we demonstrate that the shape of the formed Ag dendrites can be controlled by a combination of an applied electric field and patterned regions of a reduced GO film created by a focused laser beam. This paves the way for an alternative low-cost silver nanoparticle assembly method requiring only a low-powered laser and low voltage.

## Abstract 2

A silver dendrite assisted micro-display on a GO film, whose visibility is tunable by an externally applied potential, is created.

## Graphical abstract

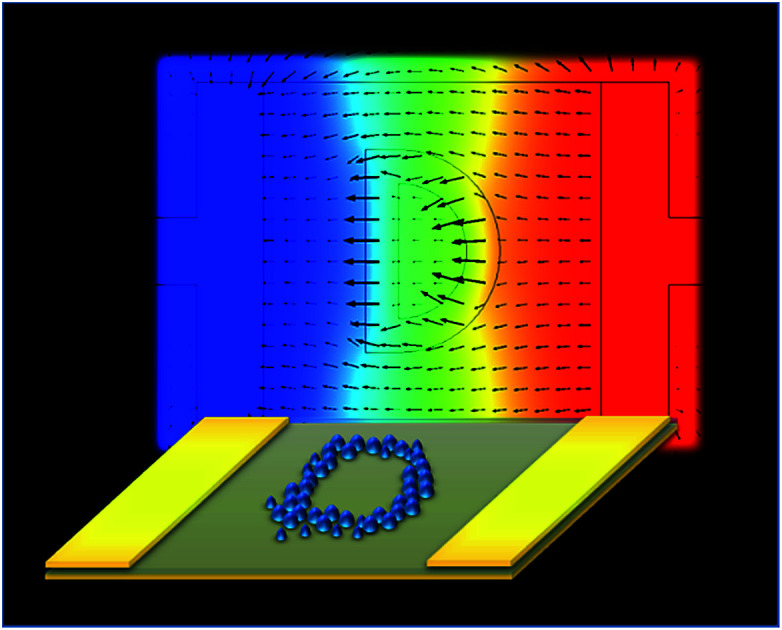

## Introduction

Metal migration, a phenomenon involving the migration of ions in the presence of moisture under electrical bias between electrodes, has been a well-studied subject since the 1970s.^1,2^ To date, it has been found that silver (Ag), which has a very low free energy of oxidation, is readily oxidized and dissolves easily in the presence of water. At the same time, the low oxidation energy also means that it can quickly be reduced to metallic silver (1) through electrodeposition at a cathode, (2) by photochemical reduction, or (3) through interaction with weak reducing agents in the presence of a medium such as moisture.^1^ As such, the effect of metal migration was found to be most prominent for silver.^2–5^ However, such a migration effect also takes place in the presence of other metals such as gold (Au)^5,6^ and copper (Cu).^4,7^ With these metals being widely used in thick interconnect devices, it serves as a strong driving force to understand and prevent the occurrence of such an effect. Interestingly, such a migration effect is not restricted to conductive surfaces; it also occurs on insulating surfaces. This is because all that is required for such migration to take place is the presence of more than a few monolayers of moisture.^8^ Unless the insulating layer is hydrophobic, it is highly likely that a thin film of moisture will form on the insulating surface, hence facilitating the migration process. Moving on, with the current trend of having devices getting smaller and thinner, it is thus important, if not critical, for us to re-visit this phenomenon and re-address its relevance in today's context.

Graphene Oxide (GO) is a 2D insulating material that can be photothermally reduced *via* a focused laser beam (FLB) technique, restoring partial conductivity in localized regions, a property which can yield applications in electronics.^9^ Silver migration is well-known in thick film ICs, and a few recent articles have highlighted the ability of Ag NPs to migrate between or within carbon materials which are in close proximity to each other. These materials range from one-dimensional carbon nanotubes to two-dimensional GO.^10,11^ In this work, we seek to further extend the current knowledge of possible interactions between these Ag NPs and the GO substrate on which they were assembled on. In the process of doing so, we aim to address, understand and add functionality to the effect of silver migration across the surface of a thin Graphene Oxide (GO) film. GO in its original form is insulating, exhibiting a sheet resistance^12^ of approximately 10^12^ Ω per sq with an energy gap in the electron density of states.^13,14^ This is mainly due to the disruption of the sp^2^ bonding networks.^15,16^ Recently, Ogata *et al.* reported detailed analyses of metal permeation into multi-layered GO.^17^ In their work, metals such as Ag and Au were observed to infuse the GO layer at the metal/GO interface in the presence of moisture. Ag permeates in the form of ions while Au takes the form of atoms during this process. It is likely that such infusion takes place through oxygenated groups and defects on the GO flakes. While their work highlighted the need to understand the interactions between metal and GO interfaces, it focused mainly on the interface between the metal coating and the supporting GO film. To have a complete understanding of the migration effect, one must consider the event occurring across the surface of the GO as well as into the GO thin film.

Beyond having fundamental understanding of why and how Ag can migrate across the GO surface, we can further functionalize the GO thin film with a focused laser beam. When subjected to a photo-thermal reduction process, a focused laser beam can locally reduce certain regions of GO with high precision to make them more electrically conductive without significantly altering their physical appearance. Reduced Graphene Oxide (rGO) can easily be modified into a state of semiconductor or a graphene-like semimetal state^13,18–22^ due to the partial restoration of the sp^2^ bonding network through incremental removal of oxygen.^15^ Given the ease with which such a reduction process can take place, laser-initiated alteration to the electronic environment on the surface of the GO film is bound to play a significant role in determining the path of migration. This process is vastly different from other reported studies,^23–25^ where laser beams are used to pattern Ag NPs by direct interaction with Ag^+^ ion solution. In our case, the substrate is “dry” and no Ag NPs are observed along the laser patterned region. Furthermore, with such controllability, GO and rGO are strong candidates to be used in future micro-electronic devices. In a recent publication, a nanoscopic non-radiative fluorescence resonance energy transfer (FRET)-light-emitting diode (LED) was reported by placing a monolayer of a transition-metal dichalcogenide (TMDC) onto silver dendrites.^26^ Given such potential application of silver dendrites, we are confident that coupled with the ability to control how these silver nanoparticles migrate, there is great potential for this patterned material to be applied in areas such as micro-displays, *etc.*

This work investigates the characteristics and cause of electrochemical Ag migration across the GO surface between two electrodes when a bias is applied. Fundamental studies accounting for the effect of GO thickness, electrode designs, and duration and magnitude of applied voltage are considered. Through such understanding, the mechanism of formation is proposed and verified through low temperature experiments. By using a focused laser beam to tune the electronic environment of the GO surface, we have achieved control over how the metallic Ag moves across the GO surface. Such an ability to control the migration path of Ag on the GO surface, on the micron-scale, has never been reported before. In doing so, we have demonstrated the possible application of this phenomenon as a micro-display.

## Experimental

### Fabrication of gold electrodes on SiO_2_/Si

The electrodes were fabricated by UV lithography and metal sputtering deposition. For the initial UV photomask with electrode patterns, a soda lime blank (Nanofilm, Westlake Village, California) with 100 nm thick chromium and a 530 nm thick layer of the AZ1518 photoresist is patterned by a direct-write laser system (Heidelberg Instruments uPG 101). Next, a 1 μm thick AZ1512 resist is deposited by spin coating and then exposed to UV light in a Mask & Bond Aligner (Karl Suss, MA8/BA6). After resist development, two layers of Cr/Au (10 nm/50 nm) are sputtered on the SiO_2_/Si substrates as an adhesion and electrode base, respectively. Finally, the lift-off process by acetone is performed to leave the electrodes on the SiO_2_/Si wafer (Nanyang Equipment Pte Ltd).

### Graphene oxide on SiO_2_/Si

Highly concentrated GO suspended in water (6 mL^−1^) is purchased from Graphene Supermarket and diluted to 2 mL^−1^. 0.25 μL of the diluted solution is deposited across oxygen treated SiO_2_/Si substrates with prefabricated gold electrodes. Using 50 sccm of oxygen to treat the patterned SiO_2_/Si substrates in a reactive ion etching chamber at 200 W for 1 min will convert the SiO_2_/Si substrate with gold electrodes to more hydrophilic. This allows the GO solution to spread more evenly across the SiO_2_/Si substrates with electrodes. Next the GO on the SiO_2_/Si substrate is heated at 60 °C on a hotplate in the ambient atmosphere until it dries.

### Silver nitrate solution

Silver nitrate solution is obtained by dissolving silver nitrate crystals (Sigma Aldrich) in de-ionized water to a concentration of 0.1 M.

### Further characterization

A JEOL JSM6700-F and Oxford Instruments X-Max^N^ 150 EDX detector are used to obtain SEM and EDX data. AFM results are obtained using a Bruker Dimension Icon. Fluorescence microscopy images (FM, Olympus BX51 Microscope) and UV, blue, green and yellow excitations are obtained using UMWU2 (300–390 nm), U-MWB2 (450–500 nm), U-MWG2 (500–560 nm) and U-MWIY2 (530–580 nm) filter cubes. Electrical measurements are obtained using a Keithley 6430 source meter.

## Results and discussion

**Fig. 1:**
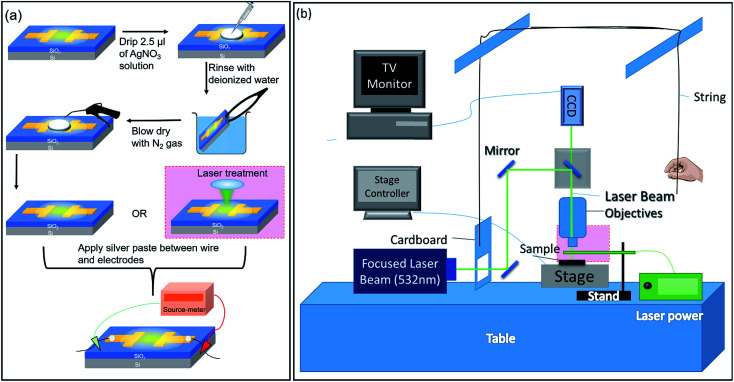
(a) Schematic of the preparation process of the AgNO_3_ treated GO samples. (b) Schematic of the focused laser beam setup.

0.1 M AgNO_3_ solution is drop-casted onto the surface of GO and rinsed with de-ionized (DI) water before it is blown dry with nitrogen gas (Fig. 1(a)). Next, the sample can be either assembled into a device or treated with a focused laser beam before being assembled into a device. A schematic depicting the focused laser beam setup is presented in Fig. 1(b). The boxed portion at the objective lens is enlarged and shown in Fig. 1(a). Using a Keithley 6430 Source Measure Unit, a potential difference is applied between the two electrodes while the sample is being observed under a fluorescence microscope (FM). In this study, the effect of (1) the distance between electrodes, (2) the magnitude of applied potential, (3) the duration of applied potential, and (4) the thickness of GO on silver migration is investigated. For each type of studies listed above, two forms of applied voltages are used: (1) sweep voltages and (2) fixed voltages at different magnitudes are applied for fixed durations. During electrical testing, the sample is observed using an FM under either Bright Field (BF) or yellow (530–580 nm) excitation. Images and videos of the observed phenomena are obtained and analyzed.

### Effect of applied potential on silver migration across the GO film

**Fig. 2:**
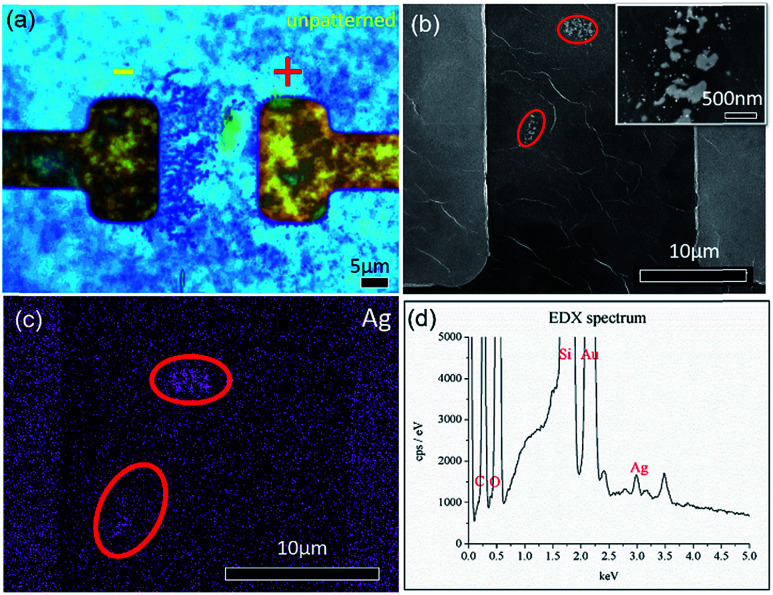
(a) BF image of dendrites formed across two gold electrodes bridged by a GO thin film. (b) SEM image of dendrites comprising small nanoparticles enclosed by the red circles. This is another sample that has undergone a similar electrical treatment to that presented in (a). The inset shows higher magnification of the region enclosed by the lower red circle in (b). (c) Elemental map of the element Ag from the region presented in (b). (d) Corresponding EDX spectra obtained from (c).

Upon applying a fixed potential of 2 V across a thin film of GO that was previously dried on a hotplate, formation of dark dendritic lines from the cathode (right electrode) towards the anode (left electrode) (Fig. 2(a)) is detected. The SEM images of similar electric field treated samples shows formation of small nanoparticles enclosed by the red circles in Fig. 2(b). The inset of Fig. 2(b) shows higher magnification of the regions enclosed by the lower red circle. From the image, two types of particles can be observed. The first type comprises sparsely distributed, small nanoparticles with an average diameter distribution of 170 ± 30 nm. The second type comprises large clusters of nanoparticles with a larger average diameter distribution of 300 ± 20 nm. This shows that the dendrites when viewed at high magnification are clusters of Ag NPs aggregated at a locale. Fig. 2(c) is an EDX elemental mapping of Ag from the sample. From the areas comprising aggregation of dendrites, a strong signal from the element Ag is detected. This confirms that the observed dendrites are Ag NPs. Fig. 2(d) shows the corresponding spectrum obtained from the region presented in Fig. 2(c).

**Fig. 3:**
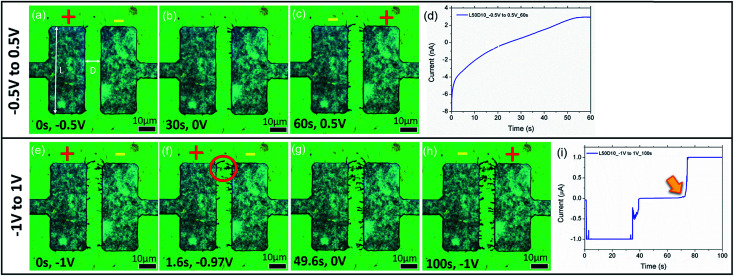
(a–c) BF time lapsed images of the sample L50D10 under a sweep voltage of −0.5 V to 0.5 V, at (a) 0 s, −0.5 V; (b) 30 s, 0 V; and (c) 60 s, 0.5 V. (d) Corresponding *I*–*t* graph to (a–c). (e–h) are time lapsed images of the same sample under a sweep voltage of −1 V to 1 V, at (e) 0 s, −1 V; (f) 1.6 s, −0.97 V; (g) 49.6 s, 0 V; and (h) 100 s, 1 V. (i) Corresponding *I*–*t* graph with dendritic ‘bridge’ formation.

To determine the effect that applied potential has on the formation of dendrites, sweep voltages of different magnitudes are applied across electrodes with a length (*L*) of 50 μm and a distance (*D*) of 10 μm (sample denoted as L50D10). Fig. 3(a–c) and (e–h) show the BF time-lapsed images of the formation of dendrites with respect to changes in the applied potential. As the potential applied increases from −0.5 V to 0 V (Fig. 3(a and b); video in the ESI, V1†), Ag^+^ ions near the cathode (right electrode) are reduced to Ag NPs through electrochemical deposition and they aggregate to form dendritic structures which grow from the cathode to the anode (left electrode). The corresponding current–time (*I*–*t*) graph is presented in Fig. 3(d). When the direction of applied voltage is reversed and increases linearly from 0 V to 0.5 V, dendrites start forming from the cathode (now the left electrode), towards the anode along the electric field (E-field) lines (Fig. 3(c); video in the ESI, V1†). Upon increasing the applied potential from −1 V to 1 V, at −0.97 V (Fig. 3(e), 1.6 s), the dendrites connect, forming a ‘bridge’ across the electrodes (enclosed by a red circle, Fig. 3(f)), resulting in a short circuit, causing a surge in the measured current (indicated by an arrow in Fig. 3(i)). To understand how the E-field can direct the growth of the dendrites, E-field lines generated across the electrodes (ESI, Fig. S1†) are simulated using COMSOL Multiphysics. Parameters used in the simulations are detailed in the ESI.† Comparing Fig. 3(h) to Fig. S1,† the dendrites show preferential growth along the E-field lines.

### Effect of electrode distance, GO thickness, and magnitude and duration of applied potential on silver migration across the GO film

**Fig. 4:**
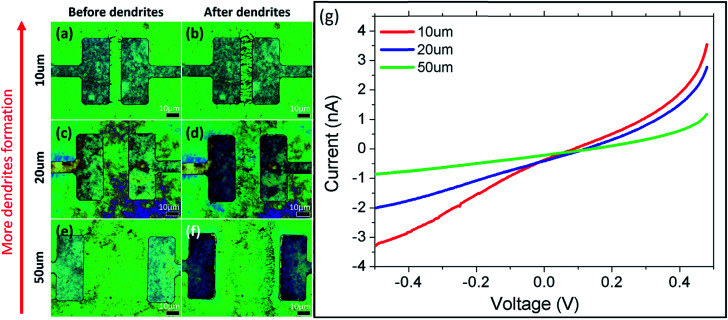
Effects of sweep voltage on the electrode distance under BF. (a–f) show the sample (a, c, e) before and (b, d, f) after electrical testing. (g) *I*–*V* graph of varying electrode distances.

Based on the above results, systematic studies involving (1) the distance between electrodes; (2) the GO thickness; (3) and the magnitude and duration of applied potential are conducted to determine how each factor influences the formation of the dendrites. In the first set of studies, lower current is detected as the distance, *D*, between the electrodes increases (Fig. 4(a–g)). This is a reasonable result because reducing the distance between electrodes decreases the resistance of the GO, thus increasing the current. At the same time, reducing the distance between the electrodes also results in an increase in electric field strength between the electrodes, which also aids in the formation of dendrites. Simulated electric field lines and strengths for electrodes with different distances are presented in Fig. S2(a–c).† This is achieved using COMSOL Multiphysics software.

In the second study, the thickness of the GO film is correlated with the observed optical color.^18^ To establish the correlation between the observed color and the thickness of the GO film, the thickness of different-colored GO samples is measured using AFM (ESI, Fig. S3†). GO colors in the order of increasing thickness are blue (56 ± 2 nm), yellow (75 ± 2 nm), dark yellow (111 ± 2 nm) and pink (127 ± 2 nm). As GO is not so conductive, thicker GO has higher resistance and thus results in lower current (ESI, Fig. S4(m)†). Hence, dendrite formation is greater on thinner (yellow, Fig. 4(a–f)) GO films compared to thicker (pink, Fig. S4(g–l)†) ones. Since time is required for the formation of dendrites to take place under an applied potential, investigation on the effect of magnitude and duration of applied potential is carried out to deepen our understanding of the mechanism.

Dendrite growth exhibits a positive relation to the magnitude of the applied potential. Potentials of different magnitudes are applied across the electrode for 30 s (†). Longer dendrite extensions can be observed with higher applied potentials. At 0.75 V (Fig. S5(a)†), no dendrites formed. At 1 V (Fig. S5(b)†), some dendrites with dark blue appearance start to form at the cathode. Upon increasing the potential to 1.25 V (Fig. S5(c)†), dendritic bridging is observed (video in the ESI, V2†). When higher potential is applied across the electrodes, higher current will flow through the GO film. With electrons flowing from the cathode to Ag^+^ ions at a higher rate, more reduction of Ag^+^ ions takes place. In the process, Ag NPs are formed. To initiate the growth of dendrites while avoiding short circuit induced by dendritic bridging, a constant potential of 1 V is applied for 20 s (Fig. S5(d)†) and 30 s (Fig. S5(e)†). Increasing the duration of applied potential provided more time for the cathode to reduce Ag^+^ ions to Ag NPs. This results in the formation of more dendrites.

### Mechanism discussion

**Fig. 5:**
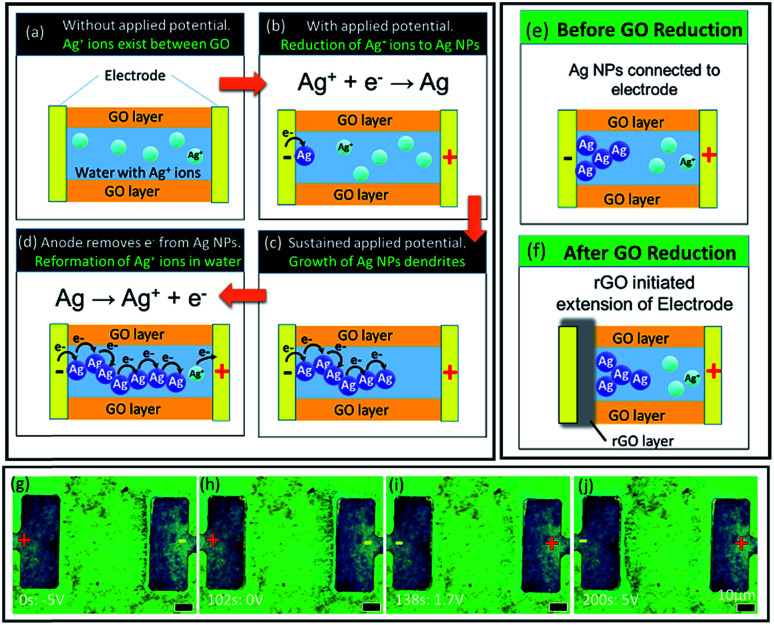
Mechanism of Ag migration. (a–d) Schematic of the mechanism. (e and f) Illustration of dendritic growth at the rGO–GO interface. (g–j) Dendritic growth on the sample L50D50 at (g) 0 s, −5 V; (h) 102 s, 0 V; (i) 138 s, 1.7 V; and (j) 200 s, 5 V.

From the above analysis, we propose that in the presence of an applied potential, formation of silver dendrites takes place through the electrochemical deposition process. During the preparation process, GO has been previously submerged in aqueous AgNO_3_ solution. As a result, water containing Ag^+^ ions is present and trapped between the GO flakes (Fig. 5(a)). This serves as the electrolyte during the electrochemical deposition process.

During the process, when a voltage is applied, electrons flow from the cathode to the anode. The Ag^+^ ions in the interlayer water take in electrons and become reduced at the interface between the water and the cathode. The reduction process resulted in the formation of Ag NPs from Ag^+^ ions as depicted in Fig. 5(b). Sustaining the applied potential provided a constant supply of electrons to the system. This allows continuous conversion of Ag^+^ ions to Ag NPs. This allows the dendrites to take the appearance as if they are “extending” from the cathode towards the anode (Fig. 5(c)). At the same time, current supplied to the anode oxidizes nearby Ag NPs back into aqueous Ag^+^ ions, causing the dendrites to “retract” or disappear when they extend too close to the anode (Fig. 5(d)). The corresponding video showing the growth and “retraction” of dendrites can be found in the ESI, V3.†

After prolonged exposure to high applied potential, the region of GO around the cathode is reduced to form reduced graphene oxide (rGO), which is more conductive (Fig. 5(f)). Under yellow excitation (Fig. S6(a)†), rGO can be identified as darker regions around the electrode. Reduction of GO is only seen at larger electrode distances for similar reasons as above, as can be seen through the lack of dark regions in samples with smaller electrode distances under yellow excitation (Fig. S6(b)†). The region of rGO around the cathode effectively serves as an extension of the electrode; hence, the Ag^+^ ions are reduced at the interface of the GO and rGO (Fig. 5(f)), rather than between the metal electrode and the GO interface (Fig. 5(e)). This leaves a gap between the dendrites and the cathode (Fig. 5(g–j)). When rGO forms over existing dendrites and a voltage is reapplied, ‘waves’ of dendrites can be observed to be formed around the cathode (indicated by orange arrows in Fig. S6(c)†).

Alternatively, if the voltage is reversed, the cathode is now the anode, and the dendrites that formed previously start to be “erased” outwards from the electrode (Fig. 5(d and e); video in the ESI, V3).† However, this “erasing” or “retraction” of dendrites is only observed at greater electrode distances (where distance *D* > 20 μm, Fig. 5(c and d)) but not when the electrode distance is small (∼10 μm, Fig. S5(a–e)†). The threshold voltage *V*_T_ for the formation of dendrites is lower for smaller electrode distances; as a result, only a small applied voltage is needed to observe formation of dendrites. This results in other anions present in the interlayer water, like OH^−^, being preferentially oxidized over Ag by the anode. At greater electrode distances, *V*_T_ increases, so the voltage applied for dendrite formation to be observed would be sufficiently high enough to oxidize Ag along with the other anions.

**Fig. 6:**
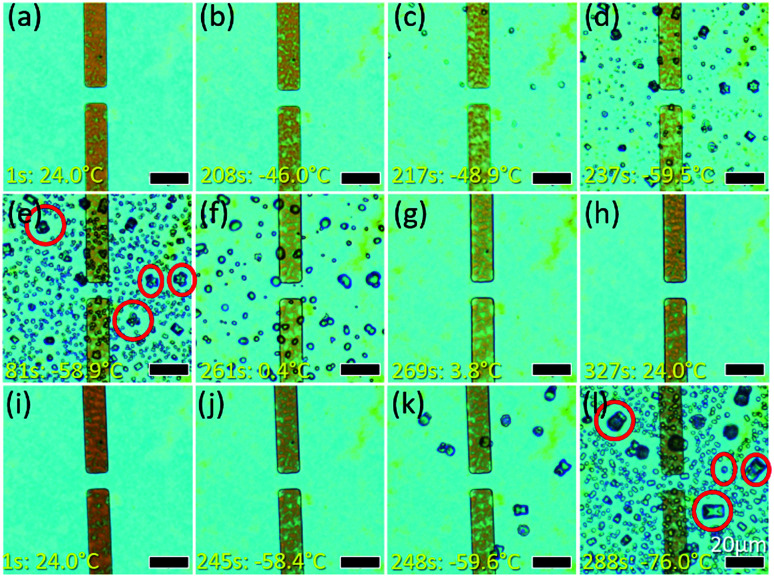
Appearance of the GO sample at different temperatures. (a) The GO initially appears blue at 24.0 °C and (b) turns yellow upon cooling to −46.0 °C. (c) Liquid droplets appear at 48.9 °C and (d) turn into crystals at −59.5 °C. (e) Crystals enlarge and more crystals form. (f) Crystals turn back into a liquid at 0.4 °C and (g) disappear at 3.8 °C (h) GO appears blue at room temperature (24 °C) again. (i–l) Second round of cooling of the same sample. (l) Crystals reform at the same position with respect to those highlighted by red circles in (e).

During the sample preparation process, the GO sample is heated at 60 °C on a hotplate in the ambient atmosphere until the solvent in which it is being dispersed dries up. As a result, one would expect the final sample to be dry. However, the presence of Ag NPs suggests that there is an interlayer electrolyte in the GO enabling the electrochemical process. To verify the presence of the suspected electrolyte – water, a controlled environment study is conducted. In this study, the GO sample is cooled using liquid nitrogen from 24 °C to −76 °C (Fig. 6(a–l); video in ESI, V4).† As the temperature decreases from room temperature to −46 °C, the GO film turned a darker hue of yellow (Fig. 6(b)). Since the optical appearance of GO is correlated with its thickness, the yellow hue observed suggested a possible increase in the thickness of the GO (ESI, Fig. S3(a and b)†). When the temperature is sufficiently low, liquid droplets form on the GO (Fig. 6(c)) and they rapidly freeze into crystals upon further cooling (Fig. 6(d and e)). Heating to 24 °C (Fig. 6(e–h)) melts the crystals into droplets (Fig. 6(f)) which are then reabsorbed into the GO film (Fig. 6(g and h); video in the ESI, V5†). A second cooling cycle (Fig. 6(i–l)) shows formation of crystals at similar sites (circled crystals in Fig. 6(e)*vs.*Fig. 6(l)). This suggests the presence of water in the GO, supported by the literature which states that there is water confined between GO layers.^1^ The thickening of the GO layer could be caused by cooling-induced expansion of water confined between the GO flakes. Eventually, water expands to a point at which it escapes through the gaps in between the GO flakes and appears as droplets which then freeze rapidly into ice crystals. This confirms that electrochemical migration of Ag NPs is possibly due to the presence of an interlayer electrolyte, water, even though GO is supposedly ‘dry’.

**Fig. 7:**
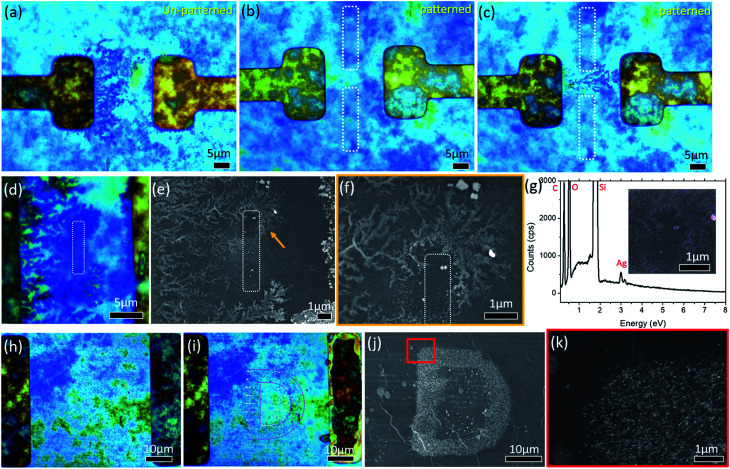
(a) Without any pre-determined reduced region on the GO film, Ag dendrites branch outwards across the entire length of the cathode towards the anode. (b) Laser initiated pre-determined reduced region between the electrodes. Patterned regions are marked by dotted rectangles. (c) Constriction of Ag dendrites through a non-reduced region on the GO film. (d) Optical and (e) SEM images of Ag dendrites bending around the laser reduced region on the GO film. The reduced region is marked by a dotted rectangle and the orange arrow highlights the bending of the Ag dendrites. (f) Higher magnification SEM image of the region indicated by an orange arrow in (e). (g) EDX spectrum and elemental map (inset) of the region in (f). (h and i) BF image of the non-reduced “D” pattern on the GO film at (h) 0 V and (i) 4 V (180 s). (j) SEM image of the “D” pattern formed using Ag NPs. (k) Higher magnification of the red square region in (j) shows site selective formation of Ag NPs.

From the above, it is evident that by controlling the distribution of the electric field on the surface of the GO film, *i.e.*, pre-determining the region of reduced GO (rGO), growth of Ag dendrites from the cathode can be choreographed along a designated path towards the anode. Such a choreographing capability is depicted in the series of images presented in Fig. 7.

In the absence of any pre-determined reduced region of the GO film (Fig. 7(a)), lines of Ag dendrites are observed to be branching outwards from the cathode towards the anode. These dendrites span across the entire length of the parallel electrode, moving along the electric field lines towards the anode (Fig. 7(a)). To control the distribution of the electric field on the GO film, site-selective reduction of the GO film between similar parallel electrodes is created through a process known as photothermal reduction. This is achieved by treating selected regions with a 532 nm focused continuous laser beam. There is no polarization plate used in the setup and the laser is set at a power of 1.75 mW and patterned at a speed of 5 μm s^−1^. A schematic of the focused laser beam setup is presented in Fig. 1(b). This creates a region with a relatively weak E-field which the Ag NPs tend to avoid. Two 20 μm long rGO lines are created between the electrodes (dotted white rectangles in Fig. 7(b)), leaving a small gap of 8 μm between the lines. In the presence of an applied potential of 2 V, Ag dendrites are observed to be constricted, avoiding the rGO regions, and pass through the gap towards the anode (Fig. 7(c)). Reversing the design, using similar laser patterning parameters, a single 5 μm line of the rGO region (dotted white rectangle in Fig. 7(d)) is created between the parallel electrodes. When a potential of 2 V is applied across the electrodes, Ag dendrites which grow in a similar manner to that presented in Fig. 7(a) are observed to avoid and bend around the rGO region before branching out again. Such choreographed bending is indicated by the orange arrow in Fig. 7(e) and a higher magnification SEM image of the indicated region is presented in Fig. 7(f). The EDX elemental map of Ag element (inset of Fig. 7(g)) and the respective spectrum (Fig. 7(g)) shows the presence of Ag element in the measured region.

Evidence shows that a focused laser beam is a viable and versatile tool, which provides us with the ability to choreograph these Ag dendrites. This in turn allows a micro-display to be achieved using this hybrid material in the presence of a small applied potential. By using the focused laser beam to raster across the entire area between the electrodes, an unaltered ‘D’ between the electrodes that is not visible under BF was left (Fig. 7(h)). After applying a constant potential of 4 V for 180 s, the letter ‘D’ becomes increasingly visible (highlighted by a pink dotted line in Fig. 7(i)). The SEM image (Fig. 7(j)) shows that the Ag NPs clustered together on the ‘D’ shaped, un-patterned region of GO. Higher magnification of the region enclosed by a red square in Fig. 7(j) is presented in Fig. 7(k). The formation of Ag NPs is restricted to the un-patterned regions with almost no Ag NPs in the patterned region.

A closer look at the laser patterned region shows the presence of a few sites with agglomeration of the Ag NPs within the laser patterned region. These sites are mainly located at regions where there is still some pristine GO. This is likely a result of the step limitation of the micro-stage which was used to move the sample with respect to the focused laser beam during the patterning process. As a result, there will be some pristine GO between each laser rastered line that will not be treated by the focused laser beam.

Apart from a micro-display, a potential application of this work lies in the viability to direct the formation of Ag dendrites into QR codes. This can be achieved with the help of the focused laser patterning process and allows a large amount of information that is pre-encrypted to be only displayed under the influence of an applied electrical potential. In the absence of the applied field or by reversing the direction of applied electrical potential, the message can be erased or smeared to the extent that the QR code is no longer visible, hence providing added security to the intended message.

## Conclusion

We have established an understanding and control of silver migration across a two-dimensional GO thin film. A mechanism involving the reduction of Ag^+^ ions in the interlayer water of GO is proposed and verified with the observation of crystal formation on a seemingly ‘dry’ GO surface under low temperature conditions. The extent of dendrite formation increased with an increase in the applied potential, an increase in the duration of applied potential, or a reduction in the electrode distance. With this understanding, a laser beam is used to alter the electric field distribution of the entire GO surface by creating site selective regions of reduced GO. This is possible as rGO is relatively more conductive than GO. Through this method, we have successfully controlled the formation of Ag NPs. By directing these NPs to a less conductive GO film, these NPs aggregate and form a shape of ‘D’. This further justifies our proposal of adding new functionality to this migration effect and using it to create a micro-display, whose visibility is tunable by an externally applied potential.

## Conflicts of interest

There are no conflicts to declare.

## Supplementary Material

NA-001-C9NA00151D-s001

NA-001-C9NA00151D-s002

NA-001-C9NA00151D-s003
