# Supplementary material for: Nanosurfer flash-mobs: electric-field-choreographed silver migration on graphene oxide
Source: Nanoscale Adv. 2019 Apr 11;1(6):2180–7. doi: 10.1039/c9na00151d (PMC9418830; doi:10.1039/c9na00151d)
Supplement: NA-001-C9NA00151D-s001 [file NA-001-C9NA00151D-s001.pdf]

Electronic Supplementary Material (ESI) for Nanoscale Advances.
This journal is © The Royal Society of Chemistry 2019

Supporting Information

Nanosurfer Flash-mobs: Electric-Field-Choreographed Silver Migration on Graphene Oxide

Yong Han Jerome Leow [§,+], *Yun Xuan Patria Lim*[§,+], *Sharon Xiaodai Lim*[^], *Jianfeng Wu* [^], *Chorng-Haur Sow*[^,*]

[§]Dunman High School, 10 Tanjong Rhu Road, Singapore 436895
[^]Department of Physics, National University of Singapore, 2 Science Drive 3, Singapore 117542

Contents of this supplementary file:

List of Videos:

Video V1. Formation of dendrites as potential applied increases from -0.5 V to 0.5 V.

Video V2. Bridging of dendrites across the electrodes.

Video V3. Growth and "retraction" of dendrites.

Video V4. Crystals formation on seemingly 'dry' GO film when cooled from 24°C to -76°C.

Video V5. Crystals disappearance on seemingly 'dry' GO film when heated from -76°C to 24°C.

List of Figures:

Figure S1. COMSOL simulation of E-field across parallel electrodes

Figure S2. COMSOL simulation of E-field across electrodes of different distances

Figure S3. AFM images quantifying thickness of different colored GO samples

Figure S4. Effect of electrode distances and GO thickness on the growth of dendrites

Figure S5. Effect of magnitude and duration of applied potential on the growth of dendrites

Figure S6. Observation of reduced GO regions and formation of dendrites "waves"

Figure S1. COMSOL simulation of E-field across parallel electrodes

COMSOL simulation is conducted in a two-dimensional plot using electrostatic mode. The electrodes are constructed using simple geometries and merged together to form the required design. The electrodes are placed in the middle of a simulated silicon wafer. A small square of GO is simulated to bridge across the two electrodes. Boundary conditions are used to ground the substrate while two separate electric potentials (-3V and 3V) are assigned to the left and right electrodes respectively. Meshing is created using extremely fine mode and electric field lines and strengths were plotted as shown below.

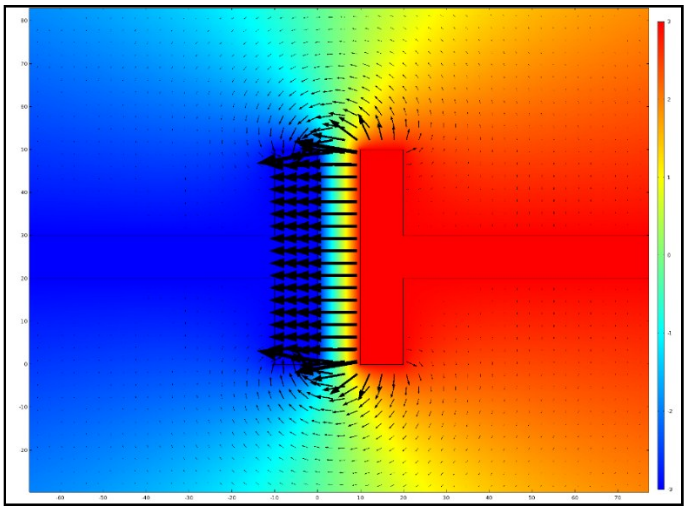

Fig.S1. COMSOL simulation of E-field between two electrodes.

Figure S2. COMSOL simulation of E-field across electrodes of different distances

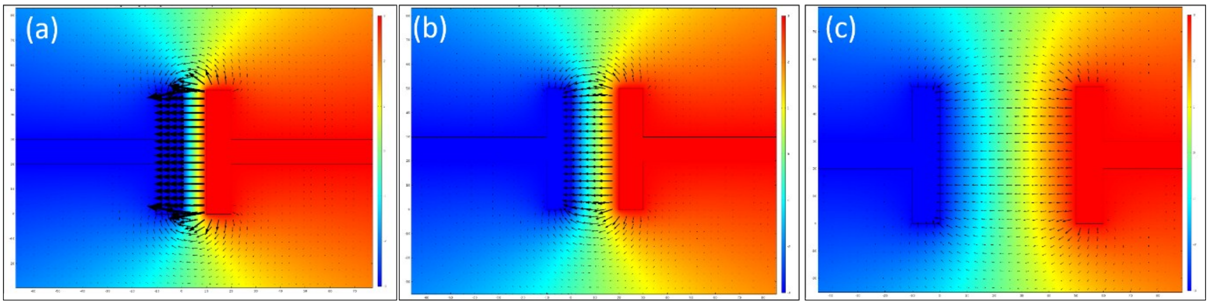

Fig.S2. Simulated electric field lines and strength for electrodes with different distances

Figure S3. AFM images quantifying thickness of different colored GO samples

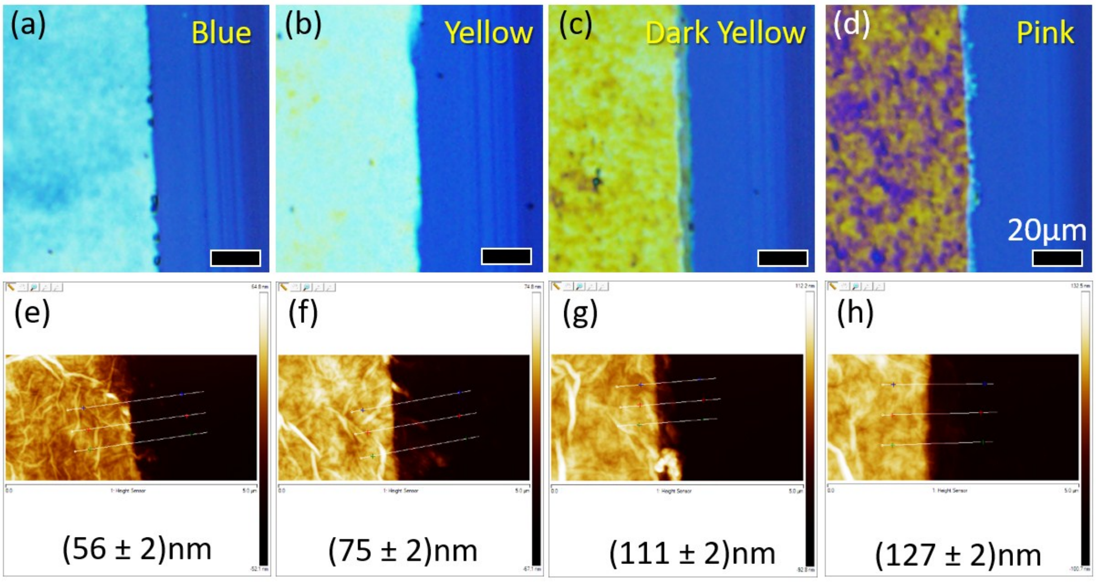

Fig.S3. AFM images analysis of GO thickness. (a-d) Optical image of (a) 56 nm-thick blue GO (b) 75 nm-thick yellow GO (c) 111 nm-thick dark yellow GO (d) 127 nm-thick pink GO. (e-h) AFM images of (a) to (d) in (e) to (h) respectively.

Figure S4. Effect of electrode distances and GO thickness on the growth of dendrites

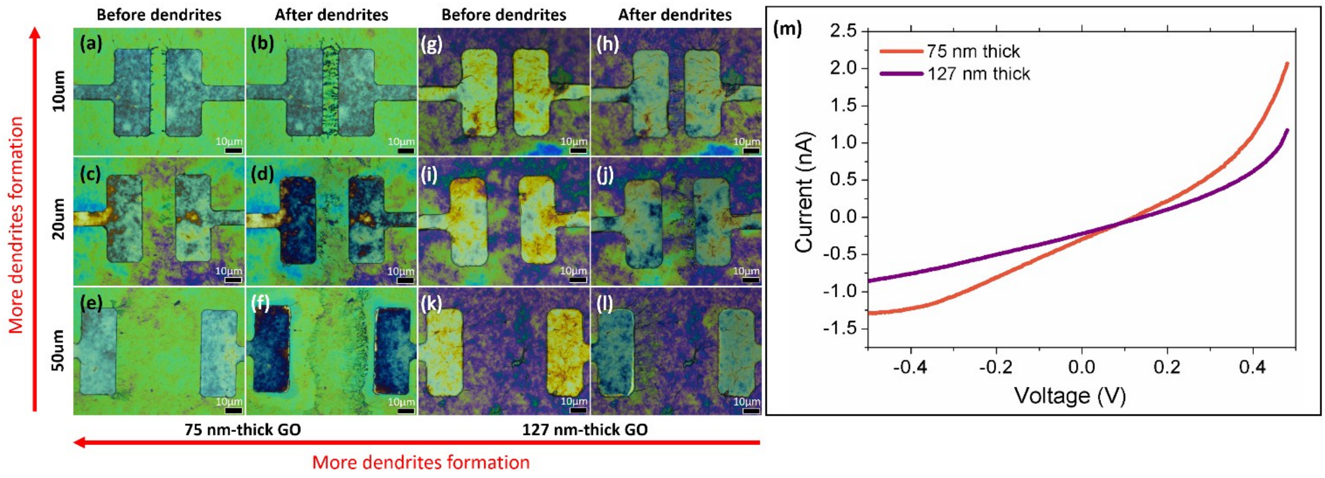

Fig.S4. Effects of sweeping voltage across electrodes of different distances and different GO thickness. (m) Compares I-V graph obtained from 75 nm and 127 nm-thick GO.

Figure S5. **Effect of magnitude and duration of applied potential on the growth of dendrites**

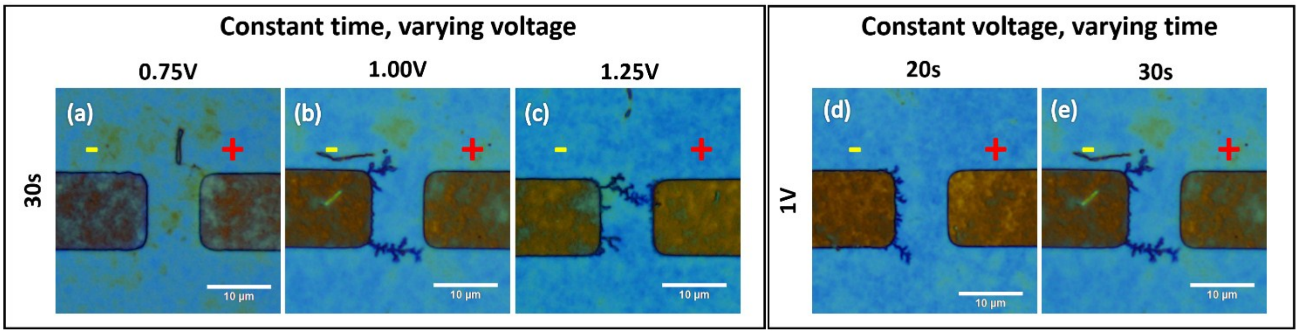

Fig.S5. (a-c) Effects of magnitude and (d-e) duration of applied potential under BF. At constant duration 30s, (a) 0.75V, (b) 1.00V, and (c) 1.25V was applied on different samples of same parameters L11D8, on 56 nm-thick GO. At constant voltage 1V, (d) 20s (e) 30s was applied on different samples of same parameters L11D8, on 56 nm-thick GO.

Figure S6. Observation of reduced GO regions and formation of dendrites "waves"

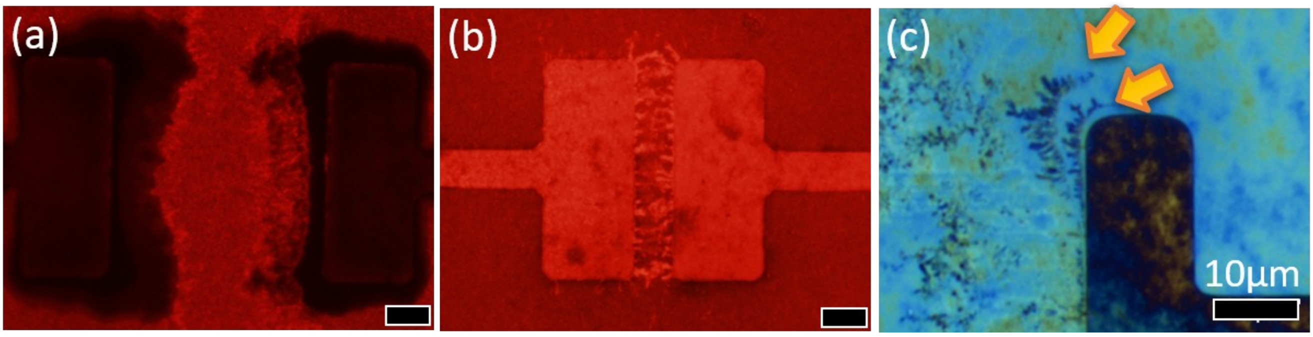

Fig.S6 (a) FM imaging of L50D50 under YF shows reduced region (darker) (b) FM imaging of L50D10 under YF shows little reduced region (i) FM BF imaging of 'waves' of dendrites forming around electrode. The waves are indicated by the orange arrows.
